# „*Je regarde l’espace différemment maintenant*.“ Ein ökogerontologischer, partizipativer Ansatz im deutsch-französischen Dialog

**DOI:** 10.1007/s00391-025-02529-y

**Published:** 2025-11-28

**Authors:** Anamaria Depner, Anna Wanka, Marion Scheider-Yilmaz, Helen Klein, Thibauld Moulaert

**Affiliations:** 1 Interdisziplinäre Alternswissenschaft , Goethe-Universität Frankfurt am Main, Frankfurt am Main, Deutschland; 2 Pacte, Université Grenoble Alpes, Grenoble, Frankreich

**Keywords:** Ökologische Gerontologie, Raum, Partizipative Forschung, Photovoice, Deutsch-Französischer Dialog, Ecological gerontology, Space, Participatory research, Photovoice, German-French dialogue

## Abstract

**Hintergrund:**

Gerontologische Theorien, Begriffe und Konzepte wandern häufig über Disziplin- und Ländergrenzen hinweg und verändern sich dabei. Literaturbasierten Untersuchungen zufolge unterscheidet sich dementsprechend das theoretische Verständnis von Raum und Altern im deutschen und im französischen Kontext.

**Fragestellung:**

Aus einer lebensweltlich orientierten Perspektive fragen wir danach, inwieweit Unterschiede im alltäglichen Raumerleben älterer Menschen in den beiden Ländern zu finden sind, und welche Dynamiken zwischen der Beziehung zum sowie der Wahrnehmung von Raum und dem Älterwerden jeweils sichtbar werden.

**Methoden:**

Mithilfe einer partizipativen Forschung mit älteren Erwachsenen, die auf der Photovoice-Methode basierte, wurde die Beziehung zwischen Raum und Altern über sprachliche, nationale und disziplinäre Grenzen hinweg erforscht.

**Ergebnisse:**

Dank der Beteiligung von älteren Co-Forschenden an der Erhebung und Analyse des Materials sowie der Theoriebildung wurde sichtbar, dass Raumbeziehungen und Raumwahrnehmungen nicht mit national-kultureller Prägung, sondern vielmehr mit Aspekten wie milieuspezifischer oder generationaler Zugehörigkeit zusammenhängen. Die Ergebnisse weisen eine Homogenität der räumlichen Wahrnehmungen und Praktiken über Ländergrenzen hinweg auf und illustrieren, wie (geteilte) Erinnerungen, gesellschaftliche Transformationsprozesse und biografische Übergänge diese prägen und verändern.

**Diskussion:**

Partizipative Ansätze haben das Potenzial, neue Perspektiven und theoretische Zugänge zu Altern und Raum zu eröffnen, die der neuen Generation älterer Menschen besser gerecht werden kann. Darüber hinaus ermöglicht eine forschungsbezogene Zusammenarbeit zwischen Wissenschaftler:innen und älteren Erwachsenen auf beiden Seiten transformative Lernerfahrungen.

## Abstract (en)

**Background:**

Gerontological theories, terms and concepts often cross disciplinary and national boundaries and change or adapt in that process. Accordingly, literature-based studies show that the theoretical understanding of space and ageing differs in the German and French contexts.

**Research question:**

From a lifeworld-oriented perspective, we ask to what extent differences in older people’s everyday experience of space can be found in the two countries and what dynamics between the relationship to as well as the personal experience of space and ageing become apparent in each case.

**Methods:**

Based on the Photovoice method the relationship between space and ageing was explored across linguistic, national and disciplinary boundaries through participatory research with older adults.

**Results:**

Thanks to the participation of older co-researchers in the collection and analysis of the material and the development of theory, it became visible that spatial relationships and spatial perception are not linked to national cultural influences but more to aspects such as milieu-specific or generational affiliation. The results show a homogeneity of spatial perceptions and practices across national borders and illustrate how (shared) memories, social transformation processes and biographical transitions shape and change them.

**Discussion:**

Participatory approaches have the potential to open up new perspectives and theoretical approaches to ageing and space that are better suited to the new generation of older adults. In addition, research-based collaboration between scientists and older adults enables transformative learning experiences on both sides.

## Hinführung zum Thema

Raumtheorien, Raumkonzept und in diesem Zusammenhang genutzte Begriffe verändern sich im Laufe der Zeit sowie über Disziplin- und Landesgrenzen hinweg. Wie aber gestalten sich das Erleben und die Wahrnehmung von „Altern und Raum“ im Lebensalltag in verschiedenen Ländern? Mithilfe eines partizipativen, in mehrerlei Hinsicht grenzüberschreitenden Vorgehens können Beziehungen zwischen Räumen und älteren Menschen gemeinsam mit diesen rekonstruiert, analysiert und interpretiert werden. Dabei wird deutlich, dass sich partizipative Ansätze nicht nur dazu eignen, Lebens- und Wohnbedingungen älterer Erwachsener zu verbessern, sondern auch dazu, (öko-)gerontologische Theorien und Konzepte weiterzuentwickeln.

## Hintergrund und Fragestellung

Die Gerontologie ist sowohl ein stark interdisziplinäres als auch international agierendes Feld. Gerontologische Theorien, Begriffe und Konzepte müssen daher häufig „auf Reisen gehen“ und sprachliche und disziplinäre Grenzen überschreiten. Zuweilen bleiben aber auch Begriffe und Theorien in benachbarten Disziplinen und Länder resonanzlos [15] oder entwickeln sich historisch selbst in benachbarten Ländern nicht deckungsgleich [16, 21]. Diese Translationen finden nicht nur innerhalb des wissenschaftlichen Feldes, sondern darüber hinaus auch in der Kommunikation mit bzw. dem aktiven Einbezug von Stakeholdern und älteren Menschen in die Forschung [z. B. 8, 10, 17] und möglicherweise auch auf ganz persönlicher Ebene im Zuge des Älterwerdens statt. Das deutsch-französische Forschungsprojekt „The Social Production of Space and Age – A French-German Dialogue towards New Theoretical Approaches and Research Pathways“ (SPAGE) geht einerseits der Frage nach, warum und wie genau sich das theoretische Verständnis von Raum und Altern im deutschen und im französischen Kontext unterscheidet, und was in den beiden länderspezifischen Diskursen jeweils voneinander gelernt werden kann.

Andererseits wird aus einer lebensweltlich orientierten Perspektive gefragt, inwieweit solche Unterschiede im alltäglichen Raumerleben älterer Menschen in den beiden Ländern zu finden sind, und, damit zusammenhängend, welche Dynamiken zwischen der Beziehung zum sowie der Wahrnehmung von Raum und dem Älterwerden jeweils sichtbar werden. Dass sich Raumbeziehungen mit dem Älterwerden verändern, ist in der ökologischen Gerontologie etabliert [6, 14, 18]. Zugleich verändern sich auch die Räume, ob private oder öffentliche, in denen wir älter werden. Da Altern und Raum in einer Wechselwirkung von menschlichen und nichtmenschlichen Aspekten einander co-konstituieren [1, 7], haben wir es hier mit einer engmaschigen Dynamik zu tun, der Rechnung getragen werden muss.

Den beiden lebensweltlich orientierten Fragen hat sich das Projekt SPAGE mit einem partizipativen Ansatz genähert. Im vorliegenden Artikel möchten wir diesen Ansatz, der die benannten co-konstitutiven Dynamiken mitbedenkt, vorstellen, die gemeinsam erarbeiteten Ergebnisse darlegen und anschließend deren Anschlussfähigkeit für die ökologische Gerontologie diskutieren.

## Design und Methode

Um die partizipative Forschung zu rahmen, entwickelte das SPAGE-Projektteam eine Struktur, die eine multilaterale Zusammenarbeit zwischen Co-Forschenden und professionell Forschenden über Ländergrenzen hinweg ermöglichte: Zwischen April und Juni 2024 wurden sowohl in Grenoble als auch in Frankfurt am Main zwei Ganztagesworkshops abgehalten. Diese wurden im Rahmen der Veranstaltungen der Universität des dritten Lebensalters (U3L) in Frankfurt am Main und ihrem Pendant, der Université Inter-Âges du Dauphiné (UIAD) in Grenoble, angeboten (lokale Workshops, in der jeweiligen Landessprache abgehalten). Der Austausch zwischen der deutschen und der französischen Gruppe der Co-Forschenden wurde bei einer anschließenden, dreitägigen Forschungsexkursion nach Straßburg ermöglicht. Die Verständigung während dieser Exkursion fand situativ auf Englisch, Französisch und Deutsch statt oder, wenn sinnvoll, via Simultanübersetzung durch eine der professionell Forschenden im SPAGE-Team (L1-Niveau in beiden Sprachen). In Grenoble nahmen 10 Personen, in Frankfurt am Main 19 Personen an den lokalen Seminaren teil. Nach Straßburg fuhren insgesamt 17 Co-Forschende (5 aus Grenoble, 12 aus Frankfurt am Main). Bei den Co-Forschenden handelte es sich um Personen im Alter zwischen 66 und 79 Jahren. Das Verhältnis von Frauen zu Männern lag in etwa bei 2:1. Dadurch, dass die lokalen Workshops als Teil des Seminarprogramms der beiden genannten Institutionen angeboten wurden, verfügten alle Personen über einen hohen Bildungsstand (Universitätsabschluss in diversen Disziplinen). Die französischen Co-Forschenden waren alle in Frankreich geboren, unter den deutschen Co-Forschenden waren 2 Personen erst im Laufe ihres Lebens nach Deutschland gezogen. Der sozioökonomische Hintergrund der mitforschenden Personen differierte, jedoch lebte zum Zeitpunkt der Forschung niemand in prekären Verhältnissen.

Der erste Seminarblock hatte Raumerfahrung und Wahrnehmung zum Thema, sowie Methoden, diese zu erheben. Aufgrund des allgemein geäußerten Interesses wurden den Co-Forschenden dabei auch die in den jeweiligen Ländern im akademischen Diskurs führenden Raumtheorien und gängige Zugänge zu Raum und Alter(n) aus unterschiedlichen Disziplinen vorgestellt und aus einer lebensweltlichen Perspektive diskutiert. Besprochen wurden etwa relationale Raumtheorien aus der Soziologie, wie jene von Löw [12] sowie klassische und aktuelle Ansätze der ökologischen Gerontologie [z. B. 9, 19]. Im französischen Kontext wurde vornehmlich das soziogeografische Raumkonzept *territoire* von u. a. Lévy und Lussault [11] oder Di Méo [5] besprochen. Im Anschluss wurden den Co-Forschenden grundlegende Methodenkenntnisse in der Photovoice-Methode vermittelt: Fotos werden zur Beantwortung einer Frage erstellt und dienen als Ausgangspunkt einer Diskussion über das Dargestellte. Diese Methode ermöglicht, die eigene Wahrnehmung und Erfahrung einer untersuchten (sozialen) Situation sichtbar zu machen und über sprachliche Mittel hinaus zu dokumentieren [13]. Mit einem begleitenden Logbuch konnten Narrative erfasst werden, die die Auswahl des Gezeigten begründen und eine zusätzliche Analysegrundlage bildeten.

In den Wochen zwischen den beiden Workshops fertigten die Co-Forschenden bis zu 3 Fotos von Orten an, „*die sie heute anders wahrnehmen, oder zu denen sie heute ein anderes Verhältnis haben als früher*“. Einer offenen Formulierungslogik folgend, wurde der Begriff *früher* bewusst unbestimmt gelassen, um eigene Vorstellungen von Zeitlichkeit miteinfließen lassen zu können und die eigene Wahrnehmung von Veränderung adressieren und reflektieren zu können. Die dazugehörigen Logbücher boten insgesamt eine DIN-A4-Seite Platz, um zu notieren, was das Bild zeigt, was die Co-Forschenden damit verbinden, und warum sie es zur Beantwortung der Fragestellung erstellt haben.

Im zweiten Seminarblock stellten sich die Co-Forschenden die mitgebrachten Fotos gegenseitig vor und werteten sie in semimoderierten Kleingruppen von 5 bis 6 Personen gemeinsam mit offenem Kodieren in Anlehnung an die Grounded Theory Method (GTM) aus. Wie im Rahmen der Photovoice-Methode vorgesehen, dienten die Bilder dabei als Ausgangspunkt einer Diskussion und – im zweiten Schritt – zur Illustration ihrer Synthese. Ziele der Diskussion waren im vorgestellten Fall eine Kategorisierung der mitgebrachten Bilder und, damit einhergehend, eine Reflexion der Raumwahrnehmung. Als Ergebnis dieser Analysen wählten die Co-Forschenden pro Gruppe 3 Bilder aus, die sie als stellvertretend für die in den jeweiligen Kleingruppen geführten Diskussionen empfanden. Damit konnten eine kollektive, über individuelle Ortsbezüge hinausgehende Analyse vollzogen und in der Folge geteilte Orientierungsrahmen sichtbar gemacht werden. Die Darlegungen zu den ausgewählten Bildern wurden wiederum in der Gesamtgruppe diskutiert. Im Rahmen der abschließenden 3‑tägigen Forschungsexkursion nach Straßburg, bei der ein Teil der Co-Forschenden aus Frankfurt und Grenoble zusammentraf, wurden die erarbeiteten Ergebnisse und Fotos diskutiert sowie eine Analyse zu Gemeinsamkeiten und Unterschieden vorgenommen. Dabei war ein Anliegen, gemeinsam mit den Co-Forschenden zu untersuchen, ob Unterschiede – entsprechend den Befunden zum akademischen Diskurs – auch in der lebensweltlichen Perspektive zu finden sind, und, wenn ja, worauf diese zurückgeführt werden können.

## Ergebnisse

**Abb. 1:**
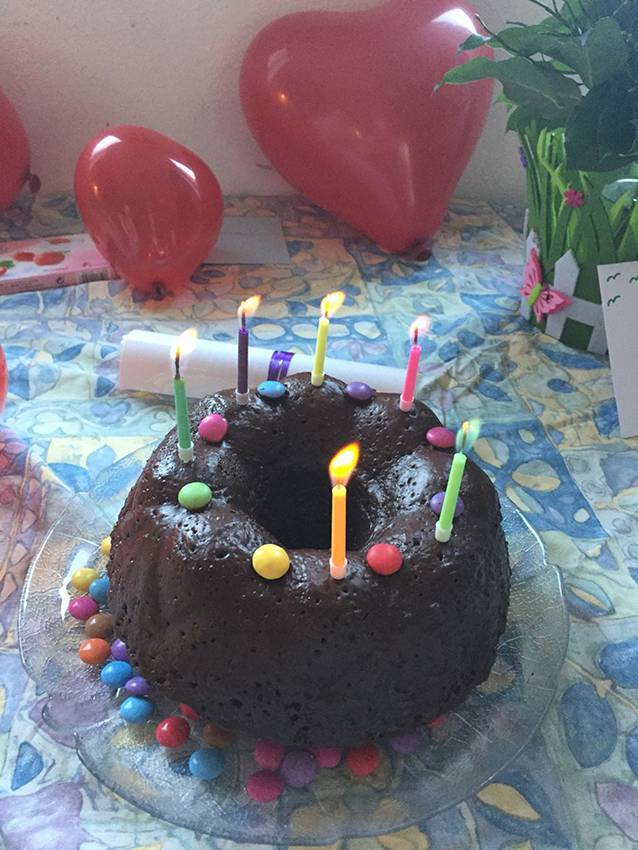
Eigene Küche als Ort für Erinnerungen, Routinen und Rituale

Die folgenden Abbildungen sind Beispiele für die in Frankfurt am Main (Abb. 1, 2 und 3) und Grenoble (Abb. 4, 5 und 6) von den Co-Forschenden erstellten und für die Diskussion ausgewählten Fotos. Sie zeigen unterschiedliche Räume, die die Co-Forschenden heute anders wahrnehmen, oder zu denen sie heute ein anderes Verhältnis haben „*als früher“*.

**Abb. 2:**
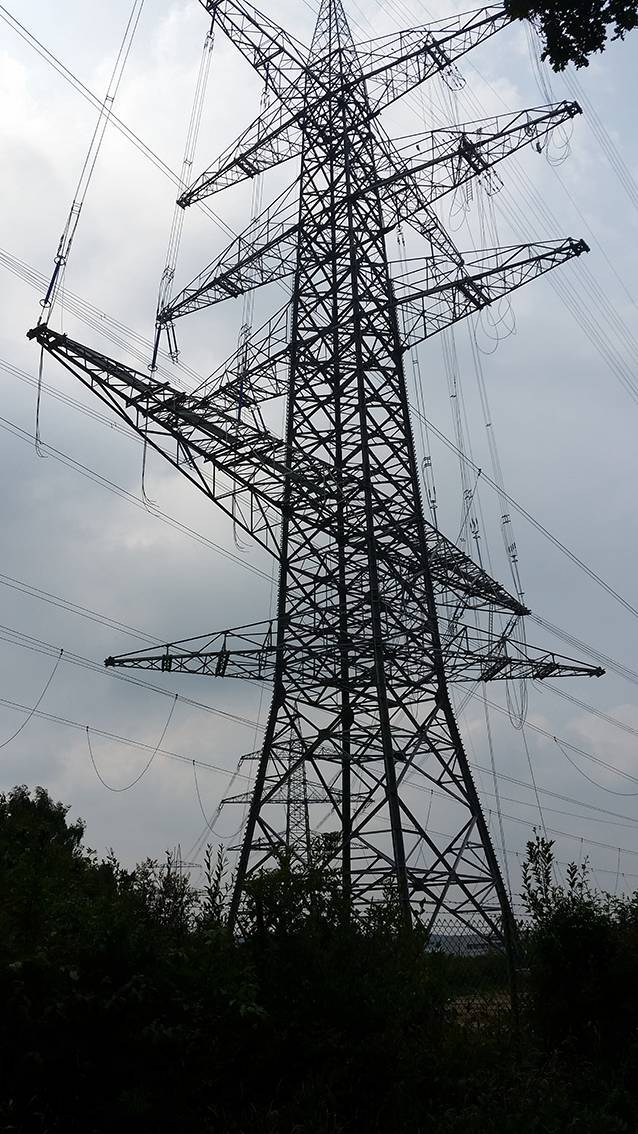
Abspannmast einer Höchstspannungsleitung, der das Landschaftsbild prägt

**Abb. 3:**
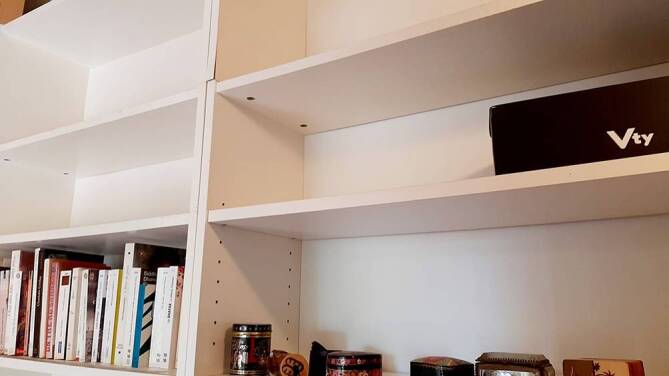
Ausgeräumtes Regal in der Wohnung

**Abb. 4:**
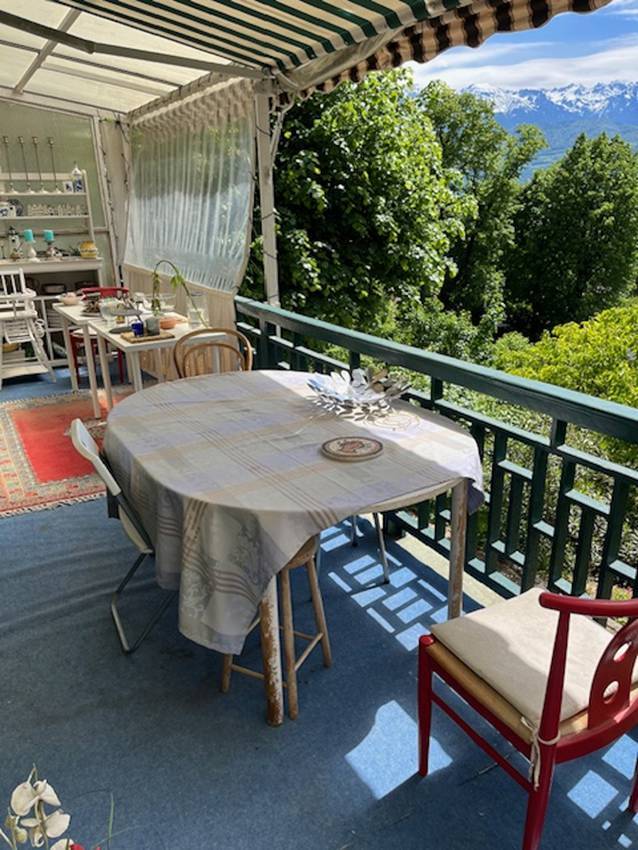
Eigene Terrasse als Ort für Erinnerungen, Routinen und Rituale

**Abb. 5:**
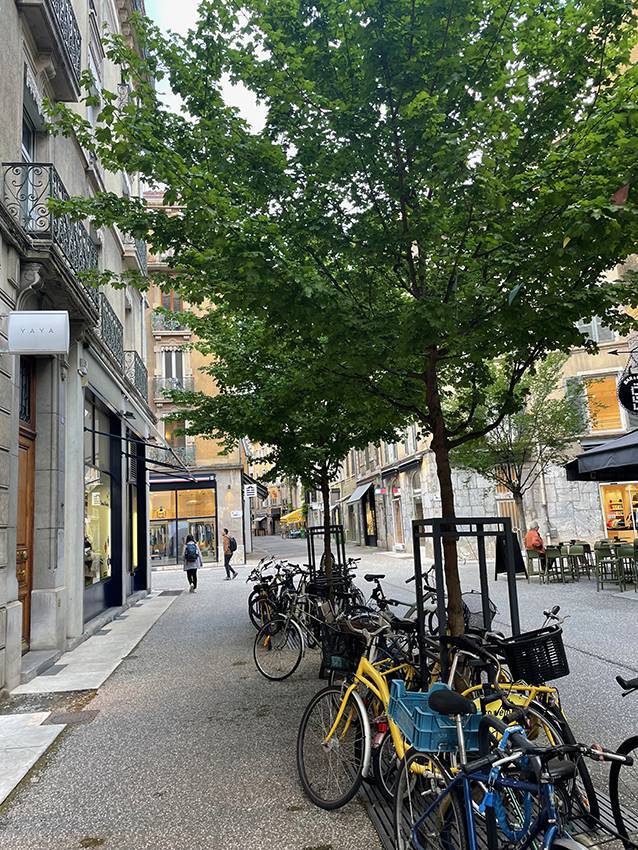
Bäume und Fahrräder an einer Stelle, an der vormals Autos verkehrten

**Abb. 6:**
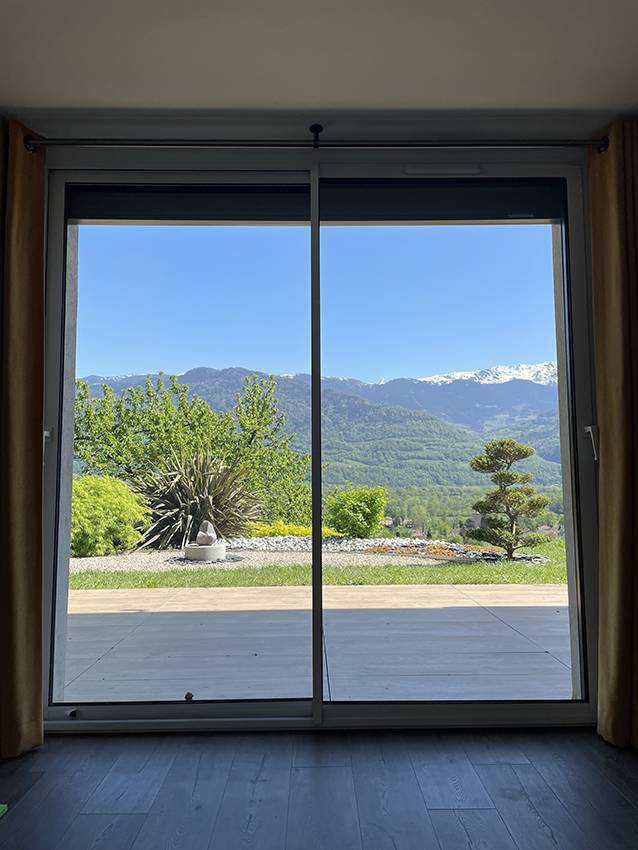
Umgebauter und umgenutzter Privatraum

Zusammen mit den Informationen aus den Logbüchern können alle von den Co-Forschenden ausgewählten Fotos, unabhängig davon, aus welchem Land sie stammen, 3 unterschiedlichen Kategorien zugeordnet werden: „Raum als Marker von Erinnerungen“ (hier exemplarisch Abb. 1 und 4), „Raum als Marker sozialer und gesellschaftlicher Transformationen“ (hier exemplarisch Abb. 2 und 5) und „Raum als Marker von biografischen Übergängen“ (hier exemplarisch Abb. 3 und 6). Diese Zuordnung wurde zuerst von den professionell Forschenden vorgenommen und anschließend mit der internationalen Gruppe von Co-Forschenden in Straßburg diskutiert und modifiziert. Ziel war es, einerseits Entwicklungen oder Veränderungen in Bezug zu und der (Be)Deutung von Raum zu erfassen, andererseits über den Vergleich der Aussagen hierzu in beiden Gruppen evtl. Gemeinsamkeiten und Unterschieden auf den Grund zu gehen. Im Folgenden hierzu einige exemplarische Einsichten in die verfassten Logbücher:

Zu einem Foto, das eine Co-Forschende in Frankfurt angefertigt hat und das in der Kategorie *Raum als Marker von Erinnerungen *eingeordnet wurde, ist im Logbuch folgende Passage zu finden:

„Der Geburtstagskuchen ist ein Ritual, das sich jedes Jahr zum Geburtstag wiederholt. So geschah es auch in meiner Kindheit. […]Dieses Ritual gebe ich gerne an meine Tochter weiter, weil es wunderschön ist, dass es sie gibt und ich sie beim Aufwachsen begleiten durfte. Der Kuchen wurde in der Küche gebacken und dort auch am Küchentisch gegessen.[…] Die Küche ist für mich auch heute noch ein besonderer Ort als Treffpunkt mit der Familie und Freunden. Das Foto symbolisiert für mich aber auch die Veränderung mit zunehmendem Alter.“

In einem Logbucheintrag aus Grenoble zu einem Foto, das in dieselbe Kategorie eingeordnet wurde, wird auf ähnliche Weise erklärt:

„Ich sah sie [meine Eltern] oft da [auf der Terrasse] sitzen, lesen oder mit ihren „alten“ Freunden sprechen. Ich hatte das Gefühl, dass sie ihre Zeit damit verschwendeten, auf dieser Terrasse zu sitzen. Was sich geändert hat, ist, dass ich ihren Platz eingenommen habe.“

In beiden Fällen werden Generationenbeziehungen angesprochen. Die Protagonist:innen, die sich in den jeweils bewohnten Küchen und auf der Terrasse finden, verändern sich im Laufe der Zeit. Gleichzeitig haben die Szenen einen Wiedererkennungswert. Die Beziehung zum gegebenen Raum und die Erinnerung an die eigene Vergangenheit sind dynamisch miteinander verbunden. Die Narrative zeigen: *Raum *kann *als Marker von Erinnerungen* fungieren, weil alltägliche Praktiken darin ihre Verortung finden oder – anders ausgedrückt – in konkreten Räumen beheimatet sind.

Wo sich *Raum als Marker für soziale und gesamtgesellschaftliche Transformationen* manifestiert, offenbaren die Logbücher kollektive Veränderungen, die das Lebensumfeld der Fotografierenden prägen. Viele Bilder zeigen städtebauliche und architektonische Entwicklungen und Veränderungen und/oder die Spuren, die politische Entscheidungen oder klimatische Ereignisse hinterlassen haben. Die hier abgebildeten Beispiele sind als Folgen menschlicher Technikentwicklung auf den Naturraum (Abb. 2) und als Folgen des menschengemachten Klimawandels (Abb. 5) vorgestellt worden.

Die Kategorie *Raum als Marker von Übergängen *umfasst jene Bilder, die biografische Veränderungen dokumentieren. Diese Ereignisse waren prägend für den Lebensverlauf und führten Veränderungen bezüglich Rollenbildern, sozialem Status, Beziehungen und Raumpraktiken mit sich. Hier zeigen viele Fotografien Veränderungen im Lebensumfeld der Co-Forschenden, wie z. B. die Umwandlung einer Garage in ein Yogastudio, nachdem das zweite Auto bei der Verrentung aufgegeben wurde (Abb. 6) oder leerstehende Regale, deren Anblick als befreiend empfunden wird, nachdem als nicht mehr benötigt identifizierte Objekte (in diesem Fall vornehmlich Bücher aus dem Arbeitskontext) aussortiert wurden (Abb. 3).

## Diskussion und Schlussfolgerungen

Inwieweit finden sich Unterschiede im alltäglichen Raumerleben älterer Menschen in der deutsch-französischen Co-Forschung?

Anders als für den historischen und den aktuellen akademischen Diskurs [12, 16, 21] gezeigt, konnten in der länderübergreifenden Analyse keine nationalen Spezifika oder Unterschiede zwischen deutschen und französischen Co-Forschenden herausgearbeitet werden. Die Gruppe setzte sich intensiv mit der Frage auseinander, worin dies begründet liegen könnte und gelangte zur erklärenden Analyse, dass es sich bei Deutschland und Frankreich um sozial, wirtschaftlich und technologisch sehr ähnlich entwickelte Länder handelt und die Co-Forschenden trotz unterschiedlicher nationaler Herkünfte eine relativ homogene Gruppe darstellten, etwa in Bezug auf Lebensphase, Generationszugehörigkeit, Bildungshintergrund und soziales Milieu.

Welche Dynamiken werden zwischen der Beziehung zum sowie der Wahrnehmung von Raum und dem Älterwerden jeweils sichtbar?

Bei aller Individualität von Lebensverläufen konnten gemeinsam mit älteren Co-Forschenden aus Deutschland und Frankreich v. a. Gemeinsamkeiten in den Beziehungen zum und den Wahrnehmungen von Raum über den Alternsprozess hinweg herausgearbeitet werden. Dabei wurde deutlich, dass Raumerfahrungen relational zur eigenen Lebenssituation und damit über die Zeit hinweg dynamisch konstituiert werden – Raum verändert sich also immer im Zusammenspiel mit biografischen Verläufen. Hier kann von einer Co-Konstitution von Raum und Altern [7, 20] gesprochen werden: So wird Raum etwa in Relation zu Übergängen im Lebenslauf verändert (z. B. die Garage umgebaut) und verändert gleichzeitig das eigene Alter(n)serleben, wenn ein Ort etwa verdeutlicht, wie Personen die Rolle der eigenen Eltern in der generationalen Ordnung einnehmen.

Mit Bezug auf Bourdieus Konzept des Habitus von sozialisierten, kollektiven und verkörperten Dispositionen, die unser Erleben, unsere Wahrnehmungen und unser Handeln in der Welt prägen [„strukturierende Erkenntniskategorien“: 2, 3] und in Verbindung mit der Co-Konstitutionsthese können wir somit davon ausgehen, dass bestimmte geteilte Sozialisationsweisen zu ähnlichem Raumerleben, ähnlichen Raumbeziehungen und ähnlichen Raumwahrnehmungen im fortgeschrittenen Erwachsenenalter führen, und dass sich diese wiederum in ähnlichen Raumpraktiken, ähnlichen Raumgestaltungs- und ähnlichen Raumaneignungsformen niederschlagen.

Vor diesem Hintergrund ist gleichzeitig davon auszugehen, dass die Untersuchung der gleichen offenen Fragestellungen bei stärker nach Milieu, Generation und sozialer Lage diversifizierten Gruppen einen Beitrag zur Theoriebildung in der Alternsforschung allgemein und speziell in der ökologischen Gerontologie leisten kann. Mit solch neuen Raumtheorien, die der dreifachen, co-konstitutiven Dynamik von Raum, Lebens(ver)lauf und vermitteltem akademischen Wissen Rechnung tragen, kann besser auf die Bedarfe einer neuen Generation älterer Menschen geantwortet werden. Dies wäre ein Ansatzpunkt für mögliche und vielversprechende zukünftige Forschungen zum Themenkomplex „Altern und Raum“, insbesondere im Hinblick auf die Untersuchung von sozialer Ungleichheit, Migrationserfahrungen oder generationsübergreifenden Perspektiven, wie Beispiele für bereits diversitätsoffene weiterentwickelte partizipative Ansätze in der Alternsforschung nahelegen [18].

Über diese wissenschaftlichen Erkenntnisse hinaus konnten wir durch das partizipative Forschen auch transformative Lernerfahrungen sowohl bei nichtakademischen als auch bei den akademischen Forschenden anstoßen. So reflektierten die Co-Forschenden, dass ihre Wahrnehmung von Raum durch die Forschung grundlegend verändert wurde. Diese Rückmeldung zeigt, dass partizipative Ansätze nicht nur weit über einen reinen Wissensgewinn oder einen Ermächtigungsaspekt hinausgehen, sondern auch neue Arten der Selbst-Welt-Beziehungen ermöglichen können. So bemerkte ein deutscher Co-Forschender: „Ich dachte, Raum sei ein Nischenthema, aber jetzt sehe ich, wie wichtig er für jeden ist, jeden Tag“, und eine französische Co-Forschende ergänzte: „Ich betrachte den Raum jetzt mit anderen Augen“ (Original: „Je regarde l’espace différemment maintenant.”). Die Co-Forschenden zeigten weiter ein großes Interesse, an der Analyse und Auseinandersetzung mit ihren sozialen und räumlichen Beziehungen zur Welt beteiligt zu werden und ihre Perspektiven einzubringen. Dies ermöglicht auch, über die manchmal zu starren Analysekategorien von professionell Forschenden hinauszugehen und sie aus einem Bezugsrahmen, der sich aus der gelebten Erfahrung der Co-Forschenden ergibt, heraus zu hinterfragen – eine Lernerfahrung, die dabei hilft, dem „scholastischen Irrtum“ entgegenzuwirken [4]. Von einem solchen spricht Bourdieu, wenn verkannt wird, dass die Primärerfahrung der Akteur:innen (Praxis) einer anderen Logik folgt als das Nachdenken über sie [4].

Die Ergebnisse der vorgestellten partizipativen Forschung sind ein überzeugendes Plädoyer für die Stärkung internationaler und partizipativer Ansätze in der Forschung, um das komplexe Zusammenspiel von Raum, Gesellschaft und Alter(n) besser zu verstehen und neue Denkansätze für die Gerontologie sowie verwandte Disziplinen zu ermöglichen.

## Fazit für die Praxis

- Partizipative Forschung fordert etablierte Machthierarchien heraus und schafft Möglichkeiten, kollektives Wissen über Raum und Alter(n) zu entwickeln.
- Durch Methoden wie *Photovoice und Logbücher* können ältere Erwachsene nicht nur als Co-Forschende aktiv eingebunden werden, sondern sie spielen auch eine bedeutende Rolle bei der Überwindung von Vorannahmen bezüglich disziplinärer, sprachlicher oder territorialer Grenzen.
- Raumbeziehungen sind aus Perspektive älter werdender Menschen sehr dynamisch. Übergänge im Lebens(ver)lauf, gesellschaftliche Transformationen und multiple Zeitlichkeiten ([erinnerte] Vergangenheit/Gegenwart/Zukunft) spielen dabei eine Rolle.
- Die verwobenen Dynamiken von Raum-Lebenslauf-Co-Konstitution müssen in die Forschungspraxis und die praktische Umsetzung von Forschungsergebnissen einbezogen werden. Differenzierungen, die entlang biografischer Aspekte und äußerer Raumveränderungen gezogen werden, helfen dabei mehr als solche, die entlang von Ländergrenzen verlaufen.

## Data Availability

Daten sind aufgrund ethischer Einschränkungen nicht verfügbar: Aufgrund der personennahen Forschungsweise sind wegen ethischer und rechtlicher Einschränkungen keine unterstützenden Daten verfügbar.
